# Evaluation proposal of a national community-based obesity prevention programme: a novel approach considering the complexity perspective

**DOI:** 10.1186/s12966-022-01271-7

**Published:** 2022-03-24

**Authors:** Irma Huiberts, Amika Singh, Frank J. van Lenthe, Mai Chinapaw, Dorine Collard

**Affiliations:** 1 Department of Public and Occupational Health, Amsterdam UMC, Amsterdam Public Health Research Institute, Vrije Universiteit Amsterdam, Van der Boechorststraat 7, 1081BT Amsterdam, The Netherlands; 2 Mulier Instituut, Utrecht, The Netherlands; 3 Center for Physically Active Learning, Faculty of Education, Arts and Sports. Western, Norway University of Applied Sciences, Sogndal, Norway; 4 Department of Public Health, Erasmus Medical Centre, Rotterdam, The Netherlands; 5 Faculty of Geosciences, Utrecht University, Utrecht, The Netherlands

**Keywords:** Community-based, Obesity, Child, Health promotion, Prevention, Evaluation, Complexity

## Abstract

Community-based obesity prevention programmes are considered an important strategy to curb the obesity epidemic. The JOGG (Youth At a Healthy Weight) approach is a large-scale community-based programme for childhood obesity prevention in the Netherlands that has been implemented over the past ten years. Practice-based development of the programme, both at the national and local level, increasingly poses challenges for its evaluation. One considerable challenge is the increasing acknowledgement of the complexity in the JOGG-approach, characterized by (a) objectives that vary locally, (b) adaptions to the programme over time in response to a community’s shifting needs, challenges and opportunities, and (c) emergent outcomes and non-linear causality.

We propose an evaluation framework that highlights elements of the complex local practice, including the local programme theory, implementation, adaption, the influence of context and feedback loops and intended as well as emergent and unintended outcomes. By studying each of these elements in practice, we hope to learn about principles that guide effective obesity prevention across contexts. The results of the proposed evaluation will inform both practice and research.

Considering complexity in evaluation is a relatively new challenge in public health and therefore an emergent research area. The proposed framework for complex evaluations allows to retrospectively evaluate a programme that was implemented and developed in practice, and enables us to learn from practice-based experiences. Following the ISBNPA Dare2Share initiative, we kindly invite other researchers in the field to share their ideas and experiences regarding integration of complexity in evaluation.

## Background

Worldwide, obesity is considered a pressing health concern, with immense consequences for population health and economy [1]. Over the past decades obesity and overweight prevalence has continued to rise [2, 3]. In order to stop this increasing trend, a large variety of programmes have been introduced to promote healthy dietary and physical activity behaviours and consequently prevent obesity [4–8].

The strategy of obesity prevention programmes in the population strongly evolved over the past decades. Early strategies were aimed at individual behaviour change [9–11]. Informed by socio-ecological frameworks of behaviour, later strategies of obesity prevention acknowledged the importance of environmental factors as drivers of unhealthy behaviour [12, 13]. Consequently, many prevention programmes implemented multiple interventions aimed at both individual and environmental drivers of obesity in multiple settings in which people live (e.g. neighbourhoods and schools). Community-based obesity prevention (CBOP) programmes [14] became a popular strategy targeting drivers of unhealthy behaviour at the local level, in which local stakeholders play an important role. Interventions in CBOP programmes are developed with and implemented by community stakeholders.

In response to the growing awareness of the complexity of obesity prevention [15], the design of CBOP programmes became increasingly complexity-oriented over the past years [16]. In this perspective, complexity refers to the context in which a programme is implemented, rather than (only) a property of the programme itself [17–20]. This context is considered a complex system of interdependent and constantly evolving elements. Within this complexity perspective it is acknowledged that the outcomes and impact of a programme are likely to vary between contexts and emerge as the programme interacts with the context. This means that even apparently simple programmes and interventions can result in varying outcomes in different local contexts [17]. The extent to which this complexity perspective is integrated within CBOP programmes ranges from dynamic programme adaptions in response the community’s changing needs, possibilities and challenges, to mapping and leveraging the characteristics of the context in order to change the whole obesity driving system [5].

Appropriate study designs for the evaluation of complex programmes are needed. Until recently, evaluation designs for CBOP programmes were commonly (quasi-) experimental designs that solely focus on the effect of the programme or specific programme components on health behaviours or obesity [21]. Experimental evaluation designs pose several challenges for complex CBOP programmes [20, 22–24], including a strong focus on predetermined and linear causal outcomes and processes, and to a lesser extent on programme development over time and context. As a consequence, much is still unknown about how CBOP programmes impact communities and obesity prevalence, and which mechanisms underly success or failure of such programmes [5, 7, 21].

In order to address these challenges and support future development of CBOP programmes, an evaluation approach is needed that considers complexity in the evaluation [20, 24, 25]. This approach requires novel types of research questions, a shift in thinking about how a programme contributes to behaviour change and obesity prevalence, and different (application of) research methods to study process and impact. So far, evaluation designs that consider complexity in its research questions and approach have been relatively uncommon in public health evaluation [20, 26, 27]. Some alternative approaches that do provide insight in working mechanisms of complex programmes and the interaction with local context, have been developed in other disciplines, including realist evaluation [28], developmental evaluation [29] and systems approaches [30, 31]. To the best of our knowledge, their use in the evaluation of CBOP programmes is still limited [21, 26].

In this paper we use a practical example of a CBOP programme to explain why alternative approaches to evaluation are sometimes more suitable than commonly used effect evaluations. In addition, we present and share our evaluation approach that considers the complexity of CBOP and actively invite other researchers to deliberate on this. By doing this we aim to contribute to the debate on how alternative evaluation approaches and methods can be applied in the evaluation of complex public health programmes.

We developed the evaluation approach presented in this paper for the evaluation of a national CBOP programme in the Netherlands, the JOGG (Youth At a Healthy Weight) approach, which has been implemented and developed in practice over the past ten years. Evaluation of this programme aims at gaining a better understanding of how the programme successfully contributes to obesity prevention in practice.

## Community-based obesity prevention: the JOGG-approach

### Background and core components of the JOGG-approach

The JOGG-approach was based on the French EPODE programme, which had showed promising results for CBOP [32, 33]. EPODE describes four core components that should be implemented in collaboration with community stakeholders: i) generating political commitment, ii) establishing public–private partnerships, iii) using social marketing techniques and iv) monitoring and evaluation [32, 34]. Each local JOGG-community uses these core components to support community engagement and collaboratively create a more ‘healthy environment’, thereby stimulating children’s healthy lifestyle in order to prevent childhood overweight and obesity. Community stakeholders are to be involved even they do not have a specific objective in public health (e.g. spatial planning sectors in local government or restaurants) with the premise that health should be considered in all policies. See van Koperen et al. [34] for the full logic model of the EPODE programme. The JOGG-organisation added the component ‘connecting prevention and care’, to encourage JOGG-communities to link interventions and stakeholders involved in the care for overweight children to overweight prevention efforts in the community.

Much is still unknown about how and for whom these EPODE-based or similar CBOP programmes work. Recent studies show mixed results [7, 8, 35–37], which may be due to the large heterogeneity between the programmes, the diversity of contexts in which they were implemented, and heterogeneity in evaluation designs and research methods. The core components of EPODE were at the time developed based on existing theories [38] and practice [34]. Few studies have since then focussed on further studying the core components or causal links between the core components and intended outcomes [21, 39].

### Organisation structure: a national JOGG-organisation and local JOGG-communities

The JOGG-approach is planned and implemented both at the national and local level, which enables influencing both macro-environmental structures (e.g. policies within the national sports sector or food sector) as well as local environmental settings (e.g. in schools and local government). The national JOGG organisation, funded by the Dutch government, creates awareness about childhood overweight and a healthy lifestyle, develops partnerships, lobbies and implements actions at the national level. In addition, the national organisation is responsible for the strategic development of the local JOGG-approach.

At the local level a JOGG-team, consisting of a local JOGG-manager and policy officer, coordinates the development and implementation of the JOGG-approach. A local action plan is developed that fits the local context, challenges and possibilities (for example local demographics or political environment). This action plan is developed in collaboration with community stakeholders (e.g. schools, welfare organisations, sports clubs or private partners).

JOGG-communities are supported by the national organisation. Support structure includes personal support from JOGG advisors and coaches, ready-made communication and campaign materials, road maps and knowledge exchange in an online portal, training sessions and network meetings. The national organisation does not provide financial support, the local JOGG-team is responsible for mobilizing their own (financial) resources.

### Development of the JOGG-approach: 10 years in practice

The JOGG-approach was first implemented in 2010 in the Netherlands as part of a national healthy weight agreement, a partnership between government, private and public partners, initiated by the ministry of Public Health, Welfare and Sport. To date, JOGG is an independent organisation and more than 170 Dutch municipalities are implementing the JOGG-approach.

Since the start in 2010, the national JOGG-organisation has continued to develop the JOGG-approach based on (1) practice-based experience in different communities, (2) knowledge exchange within the EPODE international network, and (3) collaboration with scientific partners. Over the years, the JOGG-approach developed from a multi-component programme into a CBOP programme, that builds on a socio-ecological model of health, community capacity building and recognizes the complexity of the context in which it is implemented. These changes reflect the development of CBOP programmes overall.

#### From targeting children individually to targeting children’s environment

Over time, the national JOGG-organisation gradually shifted focus from targeting the individual child to targeting children’s environment. The first years, the JOGG-approach mainly included implementing standardized social marketing materials (e.g. campaigns, events and activities that raised awareness and knowledge about health behaviours) in order to accomplish behavioural changes at the individual level. Later, the national JOGG-organisation started focussing on identifying and targeting environmental properties that drive children’s health behaviours at the local level such as schools food and drink policies and neighbourhood playgrounds. JOGG removed the core component ‘using social marketing techniques’ from their programme logic model. Creating the healthy environment became the core aim of the approach.

**Fig. 1:**
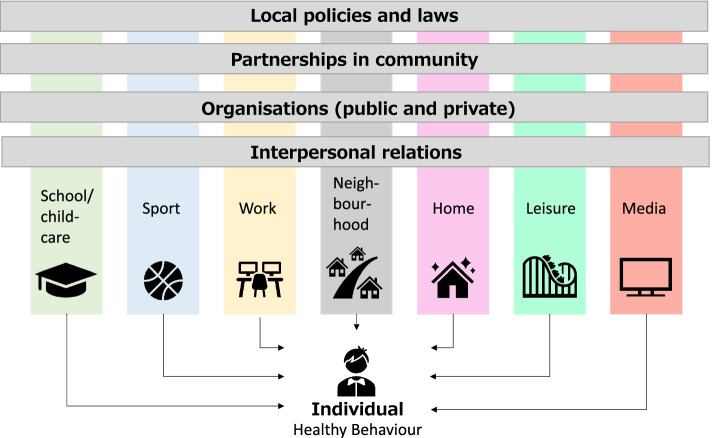
Settings and levels in the healthy environment targeted by the JOGG-approach

To date, the JOGG-organisation defines seven settings in children’s environment: family, school and child care, neighbourhood, sports clubs, leisure time settings (e.g. playgrounds or parks), work and media. The national JOGG-organisation recognises different levels of influence within these settings (see Fig. 1), which correspond with existing ecological models for health promotion [40]. The first level is the individual level: children’s behaviour, awareness, knowledge, attitudes and skills. Children’s direct social connections, for example family, peers and professionals (e.g. teachers) form the second level. The third level includes the formal and informal organisations which are present in children’s environment (e.g. schools, health services, voluntary agencies or religious organisations) and their policies and practices. The fourth level, the community level, is the community structure in which these organisations operate. For example the role of each organisation in the community, resource distribution, relations and collaborations between these organisations. This structure influences practices within the organisations, for example whether children at risk for obesity are identified and referred to fitting care. Lastly, the community and its organisations operate in a wider context of local policies and laws, the fifth level. Practices and processes on each level are inherently related and are ideally considered collectively when targeting children’s environment.

#### From collaboration in partnerships to community capacity building

From the first years onwards, stakeholders in the community (e.g. schools or food retailers) were involved in the implementation of activities, interventions and campaigns planned by the local JOGG-team [41]. Over the past five years, the JOGG-approach focus shifted to more structural community involvement through community capacity building, as shared ownership was added as a new core component in the approach. Community capacity building includes creating awareness and shared ownership of a problem in the community and providing stakeholders with support, skills and resources to develop solutions and take action together [42–44]. By building capacity among enthusiastic leaders within local stakeholder organisations in children’s environment, enthusiasm and ownership is spread, and thereby capacity for action can diffuse through whole organisations. Ideally, community stakeholders do not solely support or implement actions, they initiate, develop and manage actions from or within their own organisation, based on their insights and experiences. This ensures that the approach fits the local needs and challenges and that actions are sustained. Implementation of the JOGG-approach by the JOGG-team changed from implementing activities and interventions to community capacity building: creating a network of community stakeholders, encouraging and facilitating them to take action and maintaining an overview of the network and its (collaborative) actions to ensure coherence and contribution to the overall goals of the local approach.

#### A growing recognition of complexity

The EPODE logic model on which the JOGG-approach was based [34] illustrates a linear theory of behaviour change. Over time, the JOGG-approach integrated a growing recognition of complexity: it appeared to be not only tailored to the local context in the design phase of the local programme, it also adapted over time. The local JOGG-team is increasingly encouraged to be responsive to changing needs, opportunities, challenges and (unintended) outcomes and to adapt the approach as needed [41]. As a consequence the local JOGG-approach is continuously changing and new objectives and actions emerge over time.

In line with this, the complex causality between the JOGG-approach, the context in which it is implemented and changes they aim to bring about throughout different settings and levels of children’s environment is recognised by the national JOGG-organisation [45]. The stakeholders and organisations within these settings are not passive or isolated recipients of the JOGG-teams efforts. They exist within a wider context and interact with each other. Consequently, the causal pathway from planning and implementation to individual-level effects consists of many, unpredictable and sometimes long mutually reinforcing and non-linear cause-effect chains, that may differ across contexts.

## Evaluating the JOGG-approach

### An appropriate evaluation approach

As is the case with many other CBOP programmes [5, 7, 21], evaluations of the JOGG-approach have so far consisted of summative and formative evaluations, with research questions mainly directed at monitoring implemented activities, evaluating whether programme components were implemented as intended [46, 47] and evaluating whether the programme worked to accomplish the predefined outcomes, specifically on overweight and obesity prevalence [48–50]. Such evaluation approaches are less appropriate for the current evaluation, which aims to gain a better understanding of how the programme works in practice. First of all, because these evaluation approaches produce limited information on how the programme works. The main focus on predetermined outcomes and programme components hardly leaves room for building an understanding of why a local programme’s implementation is or is not successful and learning from practice-based programme development.

Second, past evaluations have done little justice to the inherent complexity of the JOGG-approach. Specifically, programme development at the local level and necessary adaptions during implementation were not acknowledged in evaluation. An evaluation approach and corresponding research questions that values adaptive implementation as a key component of the JOGG-approach and enables learning from local successes and failures is more suitable.

Further, the focus on solely predetermined outcomes and programme components is not sufficient. Due to the adaptive nature of the programme and varying contexts in which it is implemented, it may focus on issues or objectives that are no longer relevant and overlook unanticipated programme developments or outcomes. A broader perspective is required to consider all potential consequences of the JOGG-approach, on different levels in the community, including emerging or unintended outcomes and non-linear causal relations.

### Evaluation framework

**Fig. 2:**
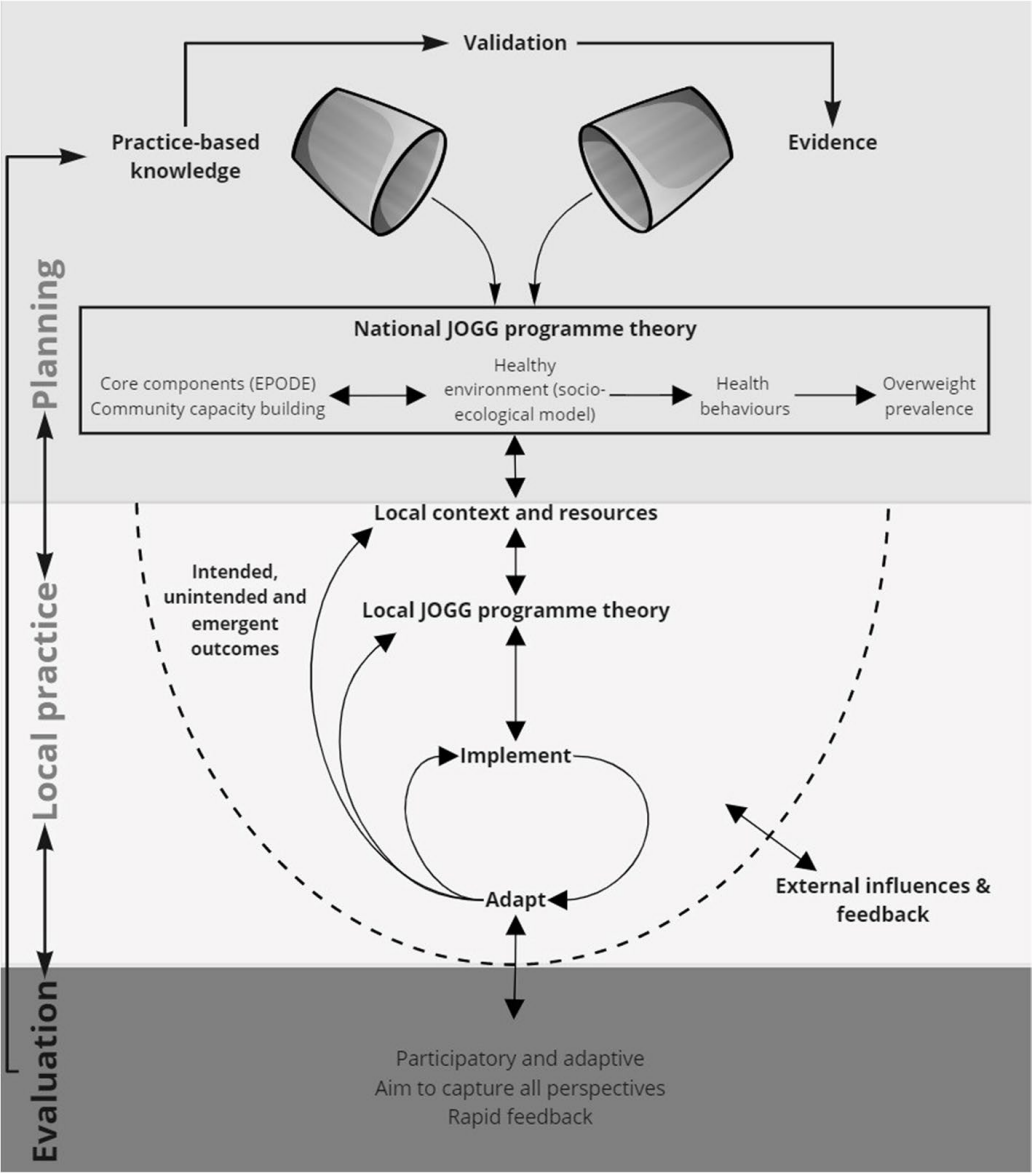
Evaluation framework of the JOGG-approach, adapted from Jolley [23]

As a foundation for the evaluation of the JOGG-approach we use the conceptual model of Jolley [23]. This model addresses the challenges of evaluating complex community-based health promotion, including the role of evaluation to support future development of a program, by building on theory based evaluation [51, 52], complexity theory [18, 53] and developmental evaluation [29]. The model provides a framework in which the JOGG-approach is recognised as a complex and adaptive process, regardless of the complexity of the programme itself (Fig. 2).

The planning phase in the model specifies the overarching JOGG programme as intended by the national JOGG-organisation, which is based on scientific evidence and practice-based knowledge. In each JOGG-community (local practice) the national programme theory is tailored to the local context and resources and is then implemented. By ‘implementation’ in this framework we mean the implementation strategies [54], all methods or techniques, that are employed by the JOGG-team to bring the programme theory into practice, in order to reach intended outcomes. This is considered a continuous and adaptive process, influenced by external factors and feedback loops. Evaluation supports both local and national developments of the JOGG-approach. Rapid feedback to stakeholders supports the adaptive implementation process at the local level. In accordance with Jolley’s model, the evaluation aims to capture perspectives of all stakeholders involved in the local approach (e.g. policy officers, private partners, professionals, target groups). Their experience and knowledge is considered a valuable source of information about the implementation process and outcomes of the local JOGG-approach. Evaluation results from the local level will feed back to the national level to support further programme development through practice-based knowledge. Compared to Jolley’s original model we added the ‘validation’ arrow from practice-based knowledge to evidence. When sufficiently validated, lessons from practice fill the ‘evidence’ bucket on which programme theories of CBOP programmes are based. Evaluation studies across different context contribute to the validation of practice-based knowledge [23, 25, 29].

### Research questions, design and methods

Research questions, design and methods for the evaluation (Table 1) are derived from the components of the model in Fig. 2 and its underlying theories. The evaluation of the JOGG-approach will consist of two phases. Phase 1 will focus on collecting knowledge from local practice. The second phase of the evaluation will then focus on validating practice-based knowledge.

**Table 1:** Research questions and methods for the evaluation of the JOGG-approach

Components framework	Research questions	Methods
National JOGG programme theory	Is the national programme theory based on the best available evidence and knowledge? Is the national programme theory likely to contribute to the intended outcomes?	Document analysis Focus groups with national programme coordinators
***Phase 1 Practice-based knowledge: multiple case studies***		
Local JOGG programme theory	What is the local programme theory (outcome goals, how goals are to be accomplished, implicit and explicit assumptions about how the programme works)? To what extend is the national programme theory translated into local programme theory? Is the local programme theory likely to contribute to the intended outcomes?	Interviews and focus groups with JOGG-team Document analysis
Local context, resources	How did the local context and local resources influence the translation of the national programme theory into the local programme theory?	Interviews and focus groups with JOGG-team
Implementation	How does the local JOGG-team implement the JOGG-approach to accomplish goals? (strategy and concrete actions)	Interviews JOGG-team Critical Event Card
Adaption	In what way did the local programme theory and the local JOGG-teams implementation strategy change and emerge over time?	Interviews JOGG-team Critical Event Card Document analysis
External influences and feedback	What key internal and external factors influenced implementation and adaption? What feedback loops influenced implementation and adaption?	Interviews JOGG-team Critical Event Card
Intended, emergent and unintended outcomes	Which planned outcomes at different levels in different settings of the healthy environment were accomplished and which were not? What outcomes emerged at different levels in different settings of the healthy environment over time? What were unintended outcomes at different levels in different settings of the healthy environment? What structural outcomes were realized at different levels in different settings of the healthy environment, and what outcomes were not sustained? What were the key internal influences, external influences and feedback loops that hindered or facilitated (structural) outcomes?	Ripple effects mapping Document analysis
Practice-based principles for effectiveness	How did the local JOGG-approach in practice (process of planning, implementation and external influence and feedback) contribute to the identified intended, emergent and unintended outcomes in the community?	Interviews JOGG-team Critical Event Card Ripple effects mapping Comparative analysis of findings in different local contexts
***Phase 2 Validating principles for effectiveness***		
Validation of principles for effectiveness	What principles guide effective action to accomplish a more healthy environment on different levels within JOGG-communities across different contexts?	To be decided

#### Phase 1 practice-based knowledge

Following the planning and implementation process in Fig. 2, the first research questions in phase 1 of the evaluation focus on the feasibility and validity of the JOGG-approach’s programme theory at both national and local level. Research questions include whether the national and (explicit or implicit) local programme theories are feasible, logical, likely contribute to childhood obesity prevention, based on the best available evidence and knowledge and what context and resource factors influenced the local translation of the national programme theory. The answers to these questions will provide insight in whether national programme theory successfully guided programme planning in local practice and whether a local programme is likely to yield results.

After this first step we address what lessons can be learned from local implementation and adaption and how they were influenced by external influences and feedback. Research questions appropriate for this step focus on what internal and external factors influenced the implementation process and in what way the local programme was adapted in response to emergent issues. The answers to these research questions will help us to unravel what (combination of) elements of the adaptive implementation process (e.g. adjustments to the approach, responses to internal or external factors) contribute to successful implementation and eventually outcomes in the community.

In order to gain insight in the impact of the JOGG-approach on the ‘healthy environment’ in the community, an essential aim in the national programme theory, the research questions focus on identifying outcomes on different levels in the healthy environment (policy, community, organisational). Intended outcomes as well as unintended and emergent outcomes are taken into account. Intended outcomes are the goals as defined in the national and local programme theory. Emergent outcomes are outcomes that emerge in response to external factors or during the implementation process, for example when new goals are developed through collaboration with stakeholders [55]. Unintended outcomes can for example occur when the response to a programme depends on the context [18]. Since the JOGG-approach aims for structural impact in the community, we specifically focus on structural outcomes of the JOGG-approach.

In the last step in phase 1 of the evaluation we focus on the extraction of practice-based principles for effectiveness, those elements of the local JOGG-approach and adaptive implementation process that contributed to the accomplished outcomes in the community. Taken together, the answers to the research questions in phase 1 provide insights in the success of the adaptive planning and implementation process, the external factors that played a role and the outcomes that were accomplished through this process. In this last step we critically review the JOGG-approach’s contribution to these outcomes. The principles of effectiveness that are identified in this phase of the evaluation fill the ‘practice-based knowledge bucket’ from which the national JOGG-organisation informs their programme theory. Validating these principles across contexts is part of phase 2 in the evaluation.

##### Design and methods

In order to answer the research questions in phase 1, we use a multiple case study design. Such a design is suitable for studying dynamic programme implementation and development within the real-life context, from multiple perspectives [56]. The large number of communities using the JOGG-approach makes it possible to do multiple case studies, considering one community as a case, and to identify principles for effectiveness that work across a range of contexts. In the multiple case studies we will use qualitative methods to answer the research questions. Qualitative methods are well suited to collect new information on what happens and why [57]. Iteratively, qualitative data will help us understand how the programme unfolded in a community and how it contributed to outcomes [58].

Research questions regarding the local programme theory will be answered applying document analysis of local programme documents and interviews with the local JOGG-team. Then, two recently developed methods will be applied to shape data collection and analysis to answer the research questions regarding implementation, adaption, external influences and outcomes, i.e. Ripple Effects Mapping [59] and the Critical Event Card [60, 61]. Both methods involve the participation of relevant stakeholders by sharing their knowledge and experiences, thereby providing insight in the complex interplay between implementation, context and outcomes. The Critical Event Card tool specifically provides insight into how and why the local JOGG-approach developed over time [60, 61], through the analysis of the critical events that characterize the evolution of the complex and adaptive programme. Ripple Effects Mapping [59] focusses on the outcomes of the JOGG-approach. This method was specifically designed to evaluate both intended and emergent outcomes of community-based programmes, across different levels in the community. It involves a group session in which different community-stakeholders participate to provide their perspective on the outcomes and collaboratively explore the contribution of the JOGG-approach.

##### Analysis: explanation building and principles for effectiveness

From each case community, practice-based principles for effectiveness will be formulated: the underlying principles that contributed to effectiveness in the local context and may provide guidance for effective action in other contexts [23, 29]. These can be formulated through the process of explanation building, which constitutes building a causal explanation about how and why certain outcomes have occurred in a case [62]. The answers to the research questions in the framework regarding local programme theory, implementation, adaption, outcomes and context, resources, external influences and feedback loops at play in each case study help build this explanation. Explanations can be visualized using a systems-based logic model [63–65], which provides insight into the complex causal relations between programme components, outcomes and their interaction (with context). By iteratively comparing explanatory propositions and the logic model from a case with findings from new cases, we expect to be able to gradually refine the model, for example by adding or unpacking components or processes [63].

The principles for effectiveness form a set of hypothesis that can be validated in the second phase of the study. Principles should eventually provide simple and pragmatic guidance for action [66] for effective community-based obesity prevention across contexts [29]. This means identifying those (combinations of) programme components that underly significant and/or structural impact in communities, for example because they set into motion a reinforcing feedback loop. To be generalizable across contexts, principles should describe the function of a programme component or action, rather than the exact form [18, 67–69]. For example, rather than formulating the action ‘organising stakeholder meetings’ a principle should provide insight in the function of this action, for example ‘building a trusting relationship with and among community stakeholders’. The exact formulation of principles for effectiveness remains to be debated and we believe it is relevant to further discuss, share ideas and experiment with different approaches. Principles could for example be formulated as simple guidance for action (e.g. ‘ensure visibility of stakeholders’ successes to other stakeholders’) or include more complex hypothesis on the causal relations between programme components, context factors and outcomes (e.g. as a causal loop diagram or parts of the systems-based logic model).

### Phase 2 validating principles for effectiveness

The second phase of the study focusses on the validation of the formulated principles for effectiveness. In this phase, additional qualitative or quantitative data may be collected. Principles may be validated using more traditional summative evaluation questions and methods. The decision on the specific methods for the second phase of the evaluation will be taken depending on the results of phase 1. The evaluation approach itself is in this way responsive to the emerging understanding of the programme through evaluation [23, 29, 68]. It remains to be debated how to adequately consider complexity in this phase of the study, especially when applying more traditional summative methods for evaluation.

## Discussion

We have proposed an evaluation approach for the JOGG-approach, a large scale community-based programme for childhood obesity prevention in the Netherlands. The evaluation of comparable programmes has been challenging, given their complexity. Which is characterized by objectives that vary locally, adaptive programme developments over time, emergent outcomes and non-linear causality. We argued that with the aim to learn about the programme components that successfully contribute to obesity prevention, the focus on only predetermined programme components and outcomes in evaluation is insufficient. The current evaluation proposal therefore focusses on the whole adaptive implementation process. The presented evaluation framework includes research questions that focus on the national and local programme theory, implementation, the interaction with local context, both intended and unintended outcomes and adaption. By focussing on these components, using complexity sensitive qualitative research methods (e.g. Ripple Effects Mapping), we aim to extract practice-based knowledge about effective principles of the programme. We expect that these principles can be used to further develop the programme and further research can focus on validating these principles for effectiveness.

 The framework will be applied retrospectively, which comes with some limitations. Indeed, it is subject to several threats to the validity, including recall bias in stakeholders and selection bias due to poor documentation or staff turnover [62]. Clearly, the risk of bias increases when the period covered in the framework increases. As some communities have implemented the JOGG-approach for over ten years, this may result in spurious causal inferences about the contribution of the JOGG-approach to outcomes in the community. In order to mitigate potential biases, several methodological elements are incorporated in the framework. First, combining data sources and methods (document analysis and interviews with different and purposively selected stakeholders) aims to increase the accuracy, as different perspectives on the same event they can be checked against each other [62]. Second, the use of timelining within the interviews helps situating participants in the past [60] and the use of the Critical Event Card tool within analyses allows to separate material evidence of events from stakeholders’ experiences and compare these [61]. Further, when identifying principles for effectiveness, cross-case analysis and explanation building are important analytical techniques to make evidence based and correct causal inferences in case studies [62]. The second study phase is explicitly aimed at validating formulated principles for effectiveness.

Despite the limitations of retrospective research, the application of the proposed framework provides valuable information. Given the fact that much is still unknown about the factors underlying success and failure of such programmes [5, 7, 21] and the impact and sustainability of these programmes in the long run [70], the expected results of the proposed evaluation, i.e. the principles for effectiveness, address some highly relevant gaps in the body of evidence of CBOP programmes. The evaluation of the JOGG-approach, which has been implemented in many different community contexts over the past 10 years, provides a unique opportunity to unravel these factors and their complex interplay (with context).

The application of a complexity perspective on the evaluation of public health programmes and interventions is not new. Over the past years different approaches and methods have been developed and applied [26, 30], for example system mapping and modelling, network analysis and the use of a complexity perspective as a lens in qualitative data analysis [58]. The proposed evaluation of the JOGG-approach however, does differ from what has been developed so far in several ways. First, existing complex evaluations mostly build on the premise that the programme was or will be designed with a complexity perspective in mind (e.g. [71, 72]). Like many other CBOP programmes however, the JOGG-approach was originally designed as a ‘simple’ programme, in which over time a greater acknowledgement of complexity was integrated. The proposed evaluation provides an opportunity to evaluate the JOGG-approach, a programme that was not specifically designed from a complexity perspective. The proposed evaluation approach may therefore be relevant for many other CBOP programmes.

Second, most existing evaluation methods and approaches that consider the complexity perspective, require to follow changes in a community system from before the start of the programme to draw conclusions about system changes over time. However, as programmes and policies are also developed in practice, without involvement of researchers in its development and implementation, other methods are also needed [25]. The proposed evaluation of the JOGG-approach is suitable to evaluate a programme that, like the JOGG-approach, was developed in practice and learn from the adaptive implementation of the programme.

Third, existing complex evaluation methods and approaches that consider the complexity perspective rarely facilitate the translation of the findings to programme planners and decision makers [26]. The proposed evaluation of the JOGG-approach is specifically aimed at generating results that inform programme planners, policy makers and health promotion practitioners to further improve the programme as well as scientist to inform the development and implementation of other CBOP programmes.

## Conclusion

The evaluation proposal described in this paper takes into account the complexity of a CBOP programme and is designed to provide insight in how the programme works. Such approaches to public health evaluation have not been given much priority in the past, and only limited guidance exists in how to apply such perspective [27]. By sharing our evaluation approach we aimed to contribute to the necessary debate and progress in this area.

Our approach is yet to be tested, and requires further specifications and developments, for example on how principles for effectiveness can best be unravelled and validated. We therefore call on other researchers to share their ideas, trials, reflections and lessons. The evaluation of complex CBOP programmes, or complex health promotion in general is not easy and relatively new, sharing ideas, approaches and lessons will support the development of appropriate and feasible evaluation approaches and methods.

## Data Availability

Not applicable.

## Abbreviations

CBOP: Community-based obesity prevention
JOGG (Jongeren Op Gezond Gewicht, Dutch): Youth At a Healthy Weight
